# Cocoa Bean Shell as a Functional Food By‐Product: Antioxidant Potential and Toxicological Thresholds in *Drosophila melanogaster*

**DOI:** 10.1002/fsn3.71861

**Published:** 2026-05-18

**Authors:** Fernanda dos Santos Trombini, Nathália Cardoso de Afonso Bonotto, Maria Eduarda Chelotti, Débora Luísa Pulcinelli, Caroline Prado Rehbein, Eliana Jardim Fernandes, Elize Musachio, Vitória Azzolin, Euler Ribeiro Filho, Maria Denise Schimith, Verônica Farina Azzolin, Ivana Beatrice Mânica da Cruz, Fernanda Barbisan

**Affiliations:** ^1^ Postgraduate Program of Nursing, Federal University of Santa Maria Santa Maria‐RS Brazil; ^2^ Postgraduate of Pharmacology, Federal University of Santa Maria Santa Maria Brazil; ^3^ Biogenomics Laboratory, Federal University of Santa Maria Santa Maria Brazil; ^4^ Postgraduate Program in Biochemistry, Federal University of Pampa Uruguaiana Brazil; ^5^ Open Third‐Age University Foundation, Amazonas Manaus Brazil; ^6^ Amazonas State University Manaus Brazil

**Keywords:** behavioral assays, bioactive compounds, dose‐dependent toxicity, *Drosophila melanogaster*, oxidative stress, phenolic compounds, polyphenols, *Theobroma cacao*

## Abstract

Agro‐industrial fruit processing generates by‐products with potential for the development of health‐promoting supplements. One such by‐product is the cocoa bean shell, a residue from chocolate production that is rich in polyphenols and other bioactive compounds. This study evaluated the chemical composition and biological effects of a combined hot and cold aqueous extract of cocoa bean shell (Cascau), using *Drosophila melanogaster* as an experimental model. Cascau was analyzed for its content of total polyphenols, flavonoids, tannins, and alkaloids, and its main constituents were identified by UPLC‐QToF‐MS. In vitro *assays confirmed moderate antioxidant activity and suggested potential genomodulatory effects, with evidence of DNA protection and no detectable DNA damage in the applied cell‐free assay*. In in vivo tests, flies were exposed to diets supplemented with different concentrations of Cascau for up to 7 days. No acute toxicity was observed after 48 h (LC_50_ = 7%, with significant effects observed at concentrations ≥ 3%). However, chronic exposure to concentrations ≥ 3% significantly reduced survival and increased oxidative stress markers and acetylcholinesterase activity, whereas locomotor function impairment was observed at higher doses. These findings indicate that Cascau exerts dose‐dependent biological effects: lower concentrations exhibit antioxidant and genoprotective potential, whereas higher concentrations induce neurotoxicity and redox imbalance. The results suggest a narrow therapeutic window and underscore the importance of dose optimization. Although Cascau displays promising bioactivity, its narrow therapeutic window suggests the need for careful evaluation prior to its application in functional foods or dietary supplements.

## Graphical abstract

The graphical abstract details the evaluation of Cascau extract, addressing its chemical composition, neurobehavioral parameters, and safety. The preparation of the CBS extract, using combined hot and cold aqueous extraction followed by lyophilization, revealed a rich chemical characterization, including polyphenols (32.04 mg GAE/g), flavonoids (10.7 mg QE/g), tannins (10.89 mg CA/g), and alkaloids (7.35 mg BNE/g). Tests in *Drosophila melanogaster* indicated an LC50 of 7% and a significant decrease in survival starting at 3%, with toxicity observed at 6%. The extract demonstrated moderate antioxidant capacity (IC50 of 104 μg/mL) and genoprotective effects, providing significant protection against H2O2‐induced DNA damage and exhibiting no genotoxicity. Regarding in vivo neurobehavioral parameters, a reduction in locomotion was observed at 5% and 6%, and an increase in climbing time at 3%, 5%, and 6%. Ex vivo analyses revealed an increase in AChE activity, ROS levels, and CAT activity at 3%, 5%, and 6%, whereas SOD activity remained at control levels (CTL). These findings underscore the potential of Cascau extract while highlighting the need to consider dosage to ensure safety and optimize its biological effects.

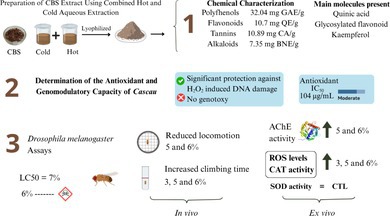

## 1 Introduction

The global food industry generates substantial quantities of agro‐industrial by‐products, many of which possess significant nutritional potential and functional properties. A notable example is cocoa bean shell, a by‐product of cocoa bean ( *Theobroma cacao* ) processing for chocolate production, which is often discarded despite its high concentration of bioactive compounds (Rojo‐Poveda et al. 2020). Recent studies have highlighted the potential of its cocoa residue as a source of dietary fiber, phenolic compounds, and methylxanthines, including theobromine and caffeine. These constituents confer antioxidant, anti‐inflammatory, and neuroprotective properties to cocoa bean shell, suggesting its applicability in the development of functional foods, nutraceuticals (Rojo‐Poveda et al. 2020; Tušek et al. 2024), and tea‐based beverages (Duque 2024).

In particular, aqueous extracts of cocoa bean shells have presented significant antioxidant activity, attributed to their high content of flavonoids and phenolic acids. For instance, Duque (2024) optimized the extraction of phenolics from cocoa bean shell, achieving noteworthy antioxidant capacity. Similarly, hydrothermal hydrolysis of cocoa bean shell has been shown to enhance the recovery of bioactive constituents, further supporting its potential for valorization.

However, despite its promising bioactive profile, it is important to emphasize that the presence of natural compounds does not inherently ensure safety or efficacy. Naturally derived substances, including those obtained from agro‐industrial residues such as cocoa bean shells, can exert complex biological effects, particularly when introduced into living organisms. In this context, cocoa bean shell contains known bioactive alkaloids, such as theobromine and caffeine, which may exert toxic effects at higher doses and can be efficiently extracted using aqueous solvents, potentially leading to their concentration in water‐based preparations. Therefore, comprehensive toxicological and pharmacological assessments are essential to determine their potential risks and therapeutic windows. Investigating the biological effects of such compounds is a fundamental step towards validating their functional claims and ensuring their safe incorporation into food products, dietary supplements, or therapeutic formulations (Delgado‐Ospina et al. 2023).

The fruit fly, *Drosophila melanogaster* , has proven to be a highly effective experimental model for toxicological and nutritional research because of its ease of handling, short life cycle, and substantial genetic similarity to vertebrates in key metabolic pathways and fundamental cellular processes. Moreover, this model enables the rapid and cost‐effective assessment of bioactive compounds on survival, behavior, and molecular markers of oxidative stress, making it an invaluable tool for the initial screening of natural substances with potential functional or therapeutic properties (Lopez‐Ortiz et al. 2023).

In light of eco‐intelligent approaches to functional food research, which underscore the importance of using non‐polluting solvents such as water (Chemat et al. 2012), a combined aqueous extract of cocoa bean shell was prepared. This extract corresponds to what is traditionally referred to as “Cascau tea”, an infusion prepared from cocoa bean shells. In the present study, the extract was obtained through a combined hot and cold aqueous extraction procedure. The effects of this extract on mortality rate, neurobehavioral parameters, and oxidative markers were evaluated using *D. melanogaster* as an experimental model. This model has previously been employed in studies involving dietary supplementation with various fruits, including Amazonian species such as açaí (Laslo et al. 2013), guaraná (Algarve et al. 2019), and camu‐camu (Musachio et al. 2024).

## 2 Methods and Materials

### 2.1 Reagents and Equipment

The extract was lyophilized using an LS3000 lyophilizer (Terroni, Lyotech, São Carlos, SP, Brazil). All reagents, except the Quant‐iT PicoGreen dsDNA Assay Kits and dsDNA reagents (Thermo Fisher Scientific, Eugene, OR, USA), were purchased from Sigma‐Aldrich (St. Louis, MO, USA). Absorbance and fluorescence measurements were carried out using a SpectraMax i3x multimode microplate reader (Molecular Devices, San Jose, CA, USA). Chromatographic analyses were performed using an ACQUITY I‐Class PLUS ultra‐performance liquid chromatography (UPLC) system (Waters Corporation, Milford, MA, USA). At the same time, mass spectrometric detection was conducted using the Xevo G2 QToF mass spectrometer (Waters Corporation, Milford, MA, USA). A refrigerated microcentrifuge (model NT 805 (X), Novatecnica, Piracicaba, SP, Brazil) was used for sample preparation.

### 2.2 Preparation of Cascau Using Combined Hot and Cold Aqueous Extraction

The cocoa bean shell, commercially available in Brazil and packaged for use as a hot and/or cold tea, was obtained from Jupará Chocolates Artesanais Co. (Salvador, BA, Brazil). Nutritional information per 100 g was provided on the product label: energy value 50 kcal, total carbohydrates (15 g), total sugars (2 g), total proteins (2 g), total fats (1 g), dietary fiber (8 g), and sodium (5 mg).

To obtain a broad spectrum of water‐soluble bioactive compounds, including polyphenols and methylxanthines, a two‐step aqueous extraction procedure combining cold and hot extraction was employed.

Initially, 200 g of cocoa bean shells were added to 2 L of chilled distilled water (4°C). The mixture was homogenized in a multiprocessor, and the pH was adjusted to 7.0 with sodium bicarbonate to minimize protein denaturation and preserve thermolabile compounds. The suspension was then stored at 4°C for 24 h. After this period, the mixture was filtered, and the resulting aqueous phase was stored in amber glass containers at 4°C to protect against light‐induced oxidation.

The solid residue obtained after filtration was subsequently subjected to a second extraction step. Two liters of distilled water heated to 80°C were added to the solid material, and the mixture was stirred on a magnetic agitator for 1 h. The suspension was then acidified with citric acid to pH 5.0, aiming to enhance the solubilization of polyphenolic compounds. After acidification, the mixture was allowed to rest at 4°C for 24 h, followed by filtration.

The aqueous extract obtained from the hot acidic extraction was then combined with the previously obtained cold extract. The pooled extract was subsequently lyophilized under controlled vacuum (180–80 μHg) and condenser temperature (−44°C) conditions, yielding a dry powdered extract, hereafter referred to as “Cascau.”

### 2.3 Chemical Characterization of Cascau

The contents of total polyphenols, flavonoids, tannins, and alkaloids in Cascau were quantified.

Total Phenolics: Quantified using the Folin–Ciocalteu method, as described by Singleton and Rossi (1965), with modifications. The reaction mixture included the sample, 0.5 mL Folin–Ciocalteu reagent, 8.5 mL Na_2_CO_3_ (7.5%), and 12 mL distilled water. After a 2‐h incubation, absorbance was measured at 765 nm. Results were expressed as mg gallic acid equivalents per gram of extract (mg GAE/g), on the basis of a gallic acid calibration curve (y = 1.8917x + 0.04; *r* ^2^ = 0.9962).

Flavonoids: Determined by the aluminium chloride (AlCl_3_) colorimetric method (Christ and Müller 1960). The reaction included methanol, sodium nitrite, AlCl_3_, sodium hydroxide, and the final volume was adjusted with methanol. After 40 min of incubation, absorbance was measured at 490 nm. Results were expressed as mg quercetin equivalents per gram of extract (mg QE/g), on the basis of a calibration curve (y = 0.8891x + 0.0327; *r* ^2^ = 0.9946).

Tannins: Quantified via the Vanillin method adapted from Morrison et al. (Morrison 1995). Tannins were extracted in 20% methanol with 1% HCl and reacted with vanillin solution. Absorbance at 490 nm was recorded after 15 min. Results were expressed as mg catechin equivalents per gram of extract (mg CE/g) using a catechin calibration curve (y = 0.0808x + 0.0343; *r* ^2^ = 0.9983).

Alkaloids: Measured using the method of Sreevidya and Mehrotra (Sreevidya and Mehrotra 2003), on the basis of the reaction of Dragendorff's reagent with nitrogen atoms in alkaloids. Results were expressed as mg bismuth nitrate equivalents per gram (mg BNE/g), with a calibration curve (y = 2.4596x + 0.0007; *r* ^2^ = 0.9945).

The identification of bioactive compounds present in Cascau was carried out by UPLC‐QToF‐MS, following a methodology adapted from Azzolin et al. (2025). Chromatographic separation was performed using an ACQUITY UPLC I‐Class PLUS system equipped with an HSS T3 column (100 Å, 1.8 μm, 2.1 × 150 mm). The mobile phases consisted of solvent A (0.1% formic acid in water) and solvent B (methanol/acetonitrile, 25:75, v/v), with a flow rate of 0.3 mL/min, column temperature of 40°C, and an injection volume of 10 μL. The gradient program was as follows: 0–1 min, 5% B; 1–10 min, 5%–40% B; 10–20 min, 40%–95% B; 20–25 min, 95% B; 25–27 min, return to 5% B, followed by re‐equilibration for 30 min.

Mass spectrometric detection was conducted in both positive and negative electrospray ionization (ESI) modes over an m/z range of 50–1200, using optimized source and acquisition parameters. Raw data were processed using Progenesis QI software, encompassing peak detection, alignment, normalization, and signal deconvolution. Compound identification was performed on the basis of the comparison of accurate mass measurements, isotopic patterns, and MS/MS fragmentation spectra with the instrument‐integrated spectral library within Progenesis QI, as well as with public databases and specialized literature.

In this step, analytical standards of the main bioactive compounds were analyzed under the same chromatographic and mass spectrometric conditions, thereby confirming compound identities by matching retention times, accurate masses, and fragmentation profiles. Identifications were assigned as putative or confirmatory according to the level of evidence available, on the basis of spectral similarity and within the mass accuracy range expected for QToF‐based high‐resolution mass spectrometry, in accordance with established guidelines for high‐resolution mass spectrometry‐based metabolomic analyses.

### 2.4 Antioxidant and Genomodulatory Capacity of Cascau

Two in vitro assays were conducted to evaluate the antioxidant and genomodulatory properties of Cascau.

Antioxidant Activity: Determined using the DPPH (2,2‐diphenyl‐1‐picrylhydrazyl) radical scavenging assay, adapted from Zhang et al. (Zhang et al. 2007). A 200 μM DPPH solution (100 μL) was combined with 100 μL of extract (1–108 μg/mL), incubated for 30 min, and read at 517 nm. The IC_50_ value was determined from the equation y = 0.4642x + 1.2798 (*r* ^2^ = 0.9728) and expressed as μg of extract (Cascau) per mL. A rutin calibration curve (1–21 μg/mL) yielded y = 3.7308x + 7.6467 (*r* ^2^ = 0.9932), which was generated to allow internal standardization and comparison with a reference antioxidant. The rutin curve was not used to express the IC_50_ in rutin equivalents but served as a positive control for assay validation.

Genomodulatory Activity: Assessed via the GEMO (genomodulatory) assay (Cadoná et al. 2014). Calf thymus dsDNA (10 μL, 20 μg/mL) was incubated with Cascau (0.1–300 μg/mL) for 30 min, then PicoGreen dye (1:200 in TE buffer) was added. Fluorescence was read after 5 min (Ex: 480 nm; Em: 520 nm). Genoprotective potential was assessed using 5 mol/L H_2_O_2_ as a pro‐oxidant control.

### 2.5 In Vivo Assessment Using the *D. melanogaster* Experimental Model

#### 2.5.1 Fly Stock and Rearing Conditions

In vivo assays using *D. melanogaster* (Harwich strain) were conducted to evaluate the safety and efficacy of Cascau. The flies used belonged to the *D. melanogaster* stock of the Biogenomics Laboratory of the Federal University of Santa Maria, RS, Brazil. They were raised under controlled conditions (25°C± 1°C; 60%–70% humidity; 12‐h light/dark cycle). The standard diet, as described by Musachio et al. (Musachio et al. 2024), consisted of 76.59% corn flour, 7.23% sugar, 8.51% wheat germ, 7.23% milk powder, 0.43% salt, and methylparaben.

#### 2.5.2 Experimental Protocol for Exposure to Cascau

**FIGURE 1:**
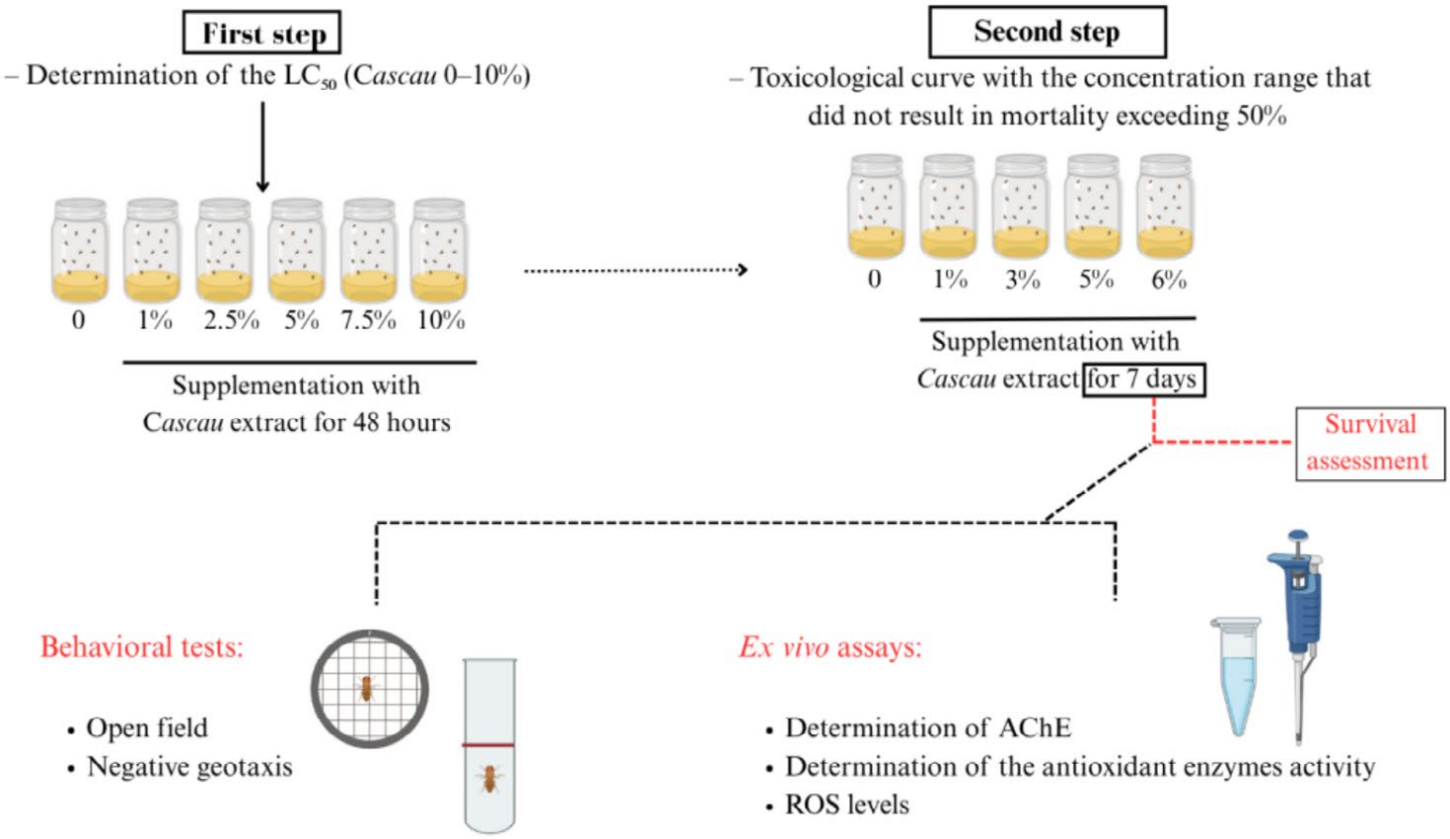
Schematic representation of the experimental methodology.

For the in vivo experimental procedure (Figure 1), each experimental group comprised 50 *D. melanogaster* flies of both sexes, aged up to 5 days. Male and female flies were used in equal proportions (1:1 both at the time of exposure and in sample preparation). Flies were randomly selected from the stock colony for each experimental replicate. Cascau supplementation was performed on a weight‐to‐weight basis (w/w), through proportional substitution of the standard diet, keeping the final mass of the preparations constant (total of 10 g of diet). The different concentrations of Cascau were added proportionally to the diet, according to the percentage of the control diet. Thus, the respective experimental groups were formed: 0% (control, without cascau addition), 1%, 3%, 5%, and 6% Cascau in 10 g of standard diet.

#### 2.5.3 Determination of LC_50_

The acute toxicity of Cascau was first evaluated by determining the concentration required to induce 50% mortality in five‐day‐old adult *D. melanogaster* (LC_50_). For this assay, groups of 50 flies were exposed for 48 h to a standard diet supplemented with varying concentrations of Cascau (0%, 1%, 2.5%, 5%, 7.5%, and 10%). The 0% concentration consisted solely of the standard fly diet, whereas the other concentrations were composed of the standard diet supplemented exclusively with the Cascau. At the end of the exposure period, both living and dead flies were counted. Four independent experiments were performed (*n* = 4).

#### 2.5.4 Survival Assessment

On the basis of the concentration range not exceeding 50% mortality, a survival assay was conducted to evaluate the long‐term effects of Cascau supplementation at concentrations of 0%, 1%, 3%, 5%, and 6%. Five independent replicates (*n* = 5) were conducted, each comprising 50 flies per group. Mortality was recorded every 24 h over a period of 7 days, and survival rates were calculated accordingly.

#### 2.5.5 Open Field Test

The open field test was employed to assess spontaneous locomotor activity and exploratory behavior in a controlled environment. This assay is pertinent for detecting potential neurobehavioral alterations induced by environmental exposures, including plant‐based extracts. The test followed the protocol of Connolly (Connolly 1966), with adaptations by Musachio et al. (Musachio et al. 2024). Briefly, five flies per group were individually transferred to a Petri dish segmented into 1 cm^2^ quadrants. After a 2‐min acclimatization period, the number of quadrants traversed within 1 min was recorded, providing a measure of locomotor performance. Four independent experiments were performed (*n* = 4).

#### 2.5.6 Negative Geotaxis Assay

Negative geotaxis evaluates motor coordination and neuromuscular function, on the basis of the innate tendency of *D. melanogaster* to climb upward following mechanical displacement. Diminished climbing performance serves as a sensitive biomarker of neuromotor impairment. The time to reach a fixed distance was used because this metric reduces the noise caused by spontaneous pauses or exploratory behaviors present in fixed‐time protocols, increasing the sensitivity to detect locomotor deficits. The test was performed as outlined by Jimenez Del‐Rio et al. (Jimenez‐Del‐Rio et al. 2010) and adapted by Paula et al. (Paula et al. 2014), using five flies per group. Each fly was placed in an individual vertical test tube and tapped gently on the bottom. The time required to climb 8 cm was recorded with a stopwatch. The test was repeated five times per fly, and individual mean climb times were calculated. Four independent experiments were performed (*n* = 4).

#### 2.5.7 Ex Vivo Assays

##### 2.5.7.1 Acetylcholinesterase (AChE) Activity

AChE activity in *D. melanogaster* serves as an established biomarker for neurotoxicity, neuroprotection, and neurodegeneration. AChE activity was quantified using the method of Ellman et al. (Ellman et al. 1961). Ten flies per group were homogenized in HEPES buffer and centrifuged at 1.000 g for 10 min. A 50 μL aliquot of the supernatant was added to 950 μL of MIX comprising 8 mL of 1 M potassium phosphate buffer, pH 8.0, 6 mL distilled water, and 2 mL of 5,5′‐dithiobis‐(2‐nitrobenzoic acid) (DTNB). Absorbance was normalized to total protein content using the Bradford assay (Bradford 1976), and the reaction was initiated with the addition of 25 μL acetylthiocholine (7.25 mM). Absorbance was measured at 412 nm, and results were expressed as μmol acetylthiocholine hydrolyzed per hour per mg of protein. Four independent experiments were performed (*n* = 4).

##### 2.5.7.2 Reactive Oxygen Species (ROS) Quantification

ROS levels were quantified using the DCFH‐DA assay, as described by Pérez‐Severiano (Pérez‐Severiano et al. 2004). Ten flies per group were homogenized in 400 μL potassium phosphate buffer (KPi) and centrifuged at 1.000 g for 5 min. A 100 μL aliquot of the supernatant was mixed with 2890 μL HEPES buffer, and the reaction was initiated by the addition of 10 μL of 1 mM DCFH‐DA. Samples were incubated for 30 min in the dark at room temperature, and fluorescence was measured at excitation and emission wavelengths of 488 nm and 520 nm, respectively. Results were expressed as percentages relative to control group values. Four independent experiments were performed (*n* = 4).

##### 2.5.7.3 Antioxidant Enzyme Activity

To determine the enzymatic activities of superoxide dismutase (SOD) and catalase (CAT), 30 flies per group were homogenized in 3.000 μL of 20 M HEPES buffer (pH 7.0). The homogenates were centrifuged at 15.000 g for 10 min; supernatants were retained for analysis. All enzyme activity values were normalized to protein concentration, determined using the Bradford assay (1976) (Bradford 1976) with bovine serum albumin (BSA) as the reference standard.

SOD Activity was assessed using the method of Kostyuk and Potapovich (1989) (Kostyuk and Potapovich 1989). The supernatant was diluted 1:10 in HEPES buffer, and 10 μL was added to a cuvette containing 1.000 μL of a reaction mixture (KPi buffer 0.025 M, pH 7.0; 0.1 mM EDTA; and TEMED). The reaction was initiated with 50 μL of quercetin. Absorbance was measured at 406 nm for 2 minutes. One unit of SOD activity was defined as the amount required to inhibit 50% of quercetin oxidation, and results were expressed in U/mg protein/min. Four independent experiments were performed (*n* = 4).

CAT Activity was measured following Aebi's method (Aebi 1984). In a quartz cuvette, 50 μL of the supernatant was added to 2.000 μL of a reaction mixture containing 0.25 M KPi buffer with 2.5 mM EDTA (pH 7.0), 30% hydrogen peroxide, distilled water, and Triton. Absorbance was recorded at 412 nm for 2 minutes. One unit of CAT was defined as the amount of enzyme required to degrade 1 μmol of H_2_O_2_ per minute. Results were expressed in U/mg protein/min. Four independent experiments were performed (*n* = 4).

### 2.6 Statistical Analysis

Power analysis (α = 0.05) was conducted on the basis of the effect size observed in the primary study to verify the adequacy of the sample size. In all experiments, *n* = 4 refers to four independent biological replicates per group. Statistical analyses and LC_50_ were conducted using GraphPad Prism version 10 (San Diego, CA, USA). Data normality and homogeneity of variances were assessed using the Shapiro–Wilk and Bartlett's tests, respectively. Comparisons between groups were performed using one‐way analysis of variance (ANOVA). When the data met the normality assumptions, Tukey's *post hoc* was used. For data that did not meet the normal distribution assumption, the Kruskal–Wallis test was used, followed by Dunn's multiple comparisons test. Results are expressed as mean ± standard deviation (SD). Statistical significance was considered at *p* ≤ 0.05.

In dose–response assays, the use of nonlinear regression with a logistic model is recommended, since the concentration‐effect relationship is biologically saturable, with minimum and maximum limits, and does not meet the assumption of constant proportionality required by linear regression (Ritz et al. 2015; Sebaugh 2011). To determine the LC_50_, the software transformed the concentrations into log_10_, and the dose–response curves were fitted by nonlinear regression using the four‐parameter logistic model (4PL). This model estimates the parameters Top, Bottom, slope (HillSlope), and logIC_50_, allowing for the precise determination of IC_50_ (inhibition concentration of 50%) from the midpoint of the fitted curve. The 4PL model is defined by the equation below, where Top and Bottom represent the upper and lower plateaus, HillSlope describes the slope of the curve, and logIC_50_ corresponds to the logarithm of the concentration that produces 50% of the effect. Thus, the LC_50_ was determined directly from the midpoint of the fitted curve.

$$\;y=\text{Bottom}+\frac{\mathrm{Top}-\text{Bottom}}{1+10^{\left(\text{logIC}50-x\right)}}\;X\;\text{HillSlope}$$

## 3 Results

### 3.1 Chemical Characterization

The polyphenol content of Cascau was 32.04 ± 0.72 mg GAE/g, flavonoids 10.7 ± 0.42 mg QE/g, tannins 10.89 ± 2.72 mg CA/g, and alkaloids 7.35 ± 0.10 mg BNE/g. The main bioactive compounds identified in *Cascau* by LC–MS/MS or HRMS are summarized in Table 1. In negative ionization mode, the presence of approximately 22–30 unique molecules was estimated, whereas in positive mode, 15–25 different bioactive compounds were detected. The 10 most abundant molecules, five from each ionization mode, on the basis of peak intensity, are listed in Table 1.

**TABLE 1:** Main putative molecules present in the Cascau obtained in the negative and positive modes identified via UPLC‐QToF‐MS.

#	Mode	Neutral mass (m/z)	Putative molecule	Relative concentration (%)**	Intensity (%)***	Fragmentation pattern (MS/MS)
1	Negative	191.0194	Quinic acid	32.6	100	Loss of H_2_O (−18 Da), loss of CO_2_ (−44 Da), fragments at m/z 127, 111
2	Negative	247.9652	Aromatic aglycone fragment (phenolic)	25.7	83	Losses of CO (−28 Da) and CO_2_ (−44 Da); aromatic ring cleavages; fragments at m/za~153–179
3	Negative	281.1115	Linoleic acid	26.8	82	Typical fatty acid fragmentation: loss of H_2_O, allylic cleavages, fragments at m/z 263, 245, 223
4	Negative	179.0344	Caffeic acid	25.7	79	Loss of CO_2_ (−44 Da), loss of H_2_O (−18 Da), fragments at m/z 135 and 107
5	Negative	353.0882	Chlorogenic acid	21.2	65	Loss of caffeoyl moiety (−162 Da), fragments at m/z 191 (quinic acid), 179 (caffeic acid)
6	Positive	412.2179	Glycosylated flavonoid*	29.3	100	Loss of sugar unit (−162 Da), fragments at m/z~271–303 (aglycones), loss of CO_2_ (−44 Da)
7	Positive	417.1757	Kaempferol 3‐O‐α‐L‐rhamnoside‐7‐O‐β‐D‐xylosyl(1 → 2)‐O‐α‐L‐rhamnoside (SGPF)	27.0	92	Loss of rhamnose (−146 Da), loss of xylose (−132 Da), fragment at m/z 287 (kaempferol aglycone)
8	Positive	377.1633	Isorhamnetin 3‐O‐rhamnoside	18.2	62	Loss of rhamnose (−146 Da), fragment at m/z 315 (isorhamnetin aglycone), methyl losses
9	Positive	307.1047	Catechin or epicatechin	13.2	45	Retro‐Diels–Alder cleavages, fragments at m/z 289, 245, 203
10	Positive	418.1782	Putative flavonoid glycoside (quercetin/kaempferol derivative)	12.3	42	Loss of sugar (−162 Da), fragments at m/z 303 (quercetin aglycone) or 287 (kaempferol), CO_2_ loss

* Possible candidate molecules: methylated kaempferol‐hexoside, acetylated quercetin‐3‐O‐rhamnoside, or dehydrated isorhamnetin‐glucoside.

** Relative concentrations (%) were estimated semi‐quantitatively from first‐stage mass spectrometry (MS^1^) peak areas obtained from extracted ion chromatograms (±5 ppm), after alignment and normalization in Progenesis QI software; values represent the normalized peak area of each compound relative to the total normalized peak area of all annotated compounds within the same electrospray ionization mode (ESI− or ESI+).

*** Intensity (%) represents base‐peak normalized signal intensity within the same mode, with the most intense ion set to 100%.

In the negative mode, the most abundant compound was quinic acid, followed by linoleic acid, caffeic acid, chlorogenic acid, and a phenolic fragment, which is likely a breakdown product of a larger phenolic structure. In the positive mode, the ion detected at m/z 412.2179 in positive ionization mode was putatively identified as a glycosylated flavonoid. On the basis of its exact mass and typical fragmentation patterns reported in the literature, this compound is likely to correspond to a methylated kaempferol‐hexoside, an acetylated quercetin‐3‐O‐rhamnoside, or a dehydrated isorhamnetin‐glucoside.

Other prominent constituents included a compound tentatively identified as kaempferol 3‐O‐α‐L‐rhamnoside‐7‐O‐β‐D‐xylosyl(1 → 2)‐O‐α‐L‐rhamnoside (SGPF), isorhamnetin 3‐O‐rhamnoside, catechin or epicatechin, and a flavonoid derivative likely related to quercetin or kaempferol. Overall, the data reveal a chemically diverse profile composed mainly of phenolic acids, flavonoids, and unsaturated fatty acids, suggesting the presence of multiple bioactive constituents with potential pharmacological relevance.

### 3.2 Antioxidant and Genoprotective Capacities

**FIGURE 2:**
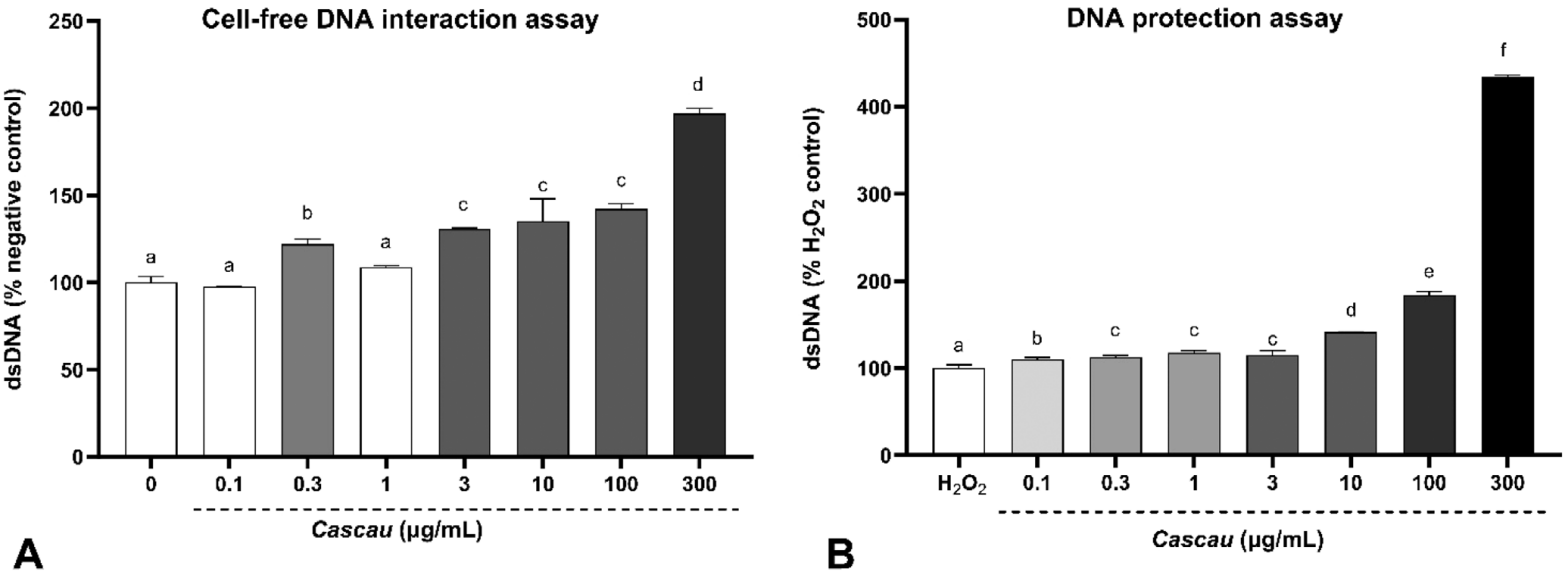
(A) Assessment of the genomodulatory effects of Cascau extract (0.1–300 μg/mL). Cell‐free DNA interaction assay: Fluorescence of calf thymus double‐stranded DNA (dsDNA) bound to the PicoGreen dye, expressed as percentage relative to the negative control group. Variations in fluorescence intensity reflect changes in dsDNA integrity or interactions affecting dye binding, which may result from DNA conformational changes or extract–DNA interactions rather than direct strand breakage. (B) DNA protection assay: DsDNA fluorescence expressed as percentage relative to the positive control group treated with 5 M H_2_O_2_, a reactive oxygen species that induces extensive DNA damage. Lower fluorescence values indicate reduced dsDNA availability for PicoGreen binding, consistent with greater DNA degradation. Data are expressed as mean ± standard deviation (SD) from four independent experiments (*n* = 4). Treatments were were statistically compared using one‐way analysis of variance (ANOVA) followed by Tukey's post hoc test. Different letters above bars indicate statistically significant differences at *p* ≤ 0.05. Groups sharing the letter “a” are not significantly different from the respective control. Lower fluorescence values indicate greater dsDNA degradation.

The concentration of a 0.5% Cascau solution required to inhibit 50% of the DPPH radical was calculated to be 104.96 μg/mL. This result suggests that Cascau exhibits a moderate level of antioxidant activity when compared with a well‐established antioxidant, the isolated rutin molecule (IC_50_ = 11.35 μg/mL). The potential genotoxic and genoprotective properties of the Cascau were evaluated using a cell‐free system consisting of purified calf thymus double‐stranded DNA (dsDNA) and PicoGreen fluorescence detection. In the assay designed to evaluate potential genotoxic effects in a cell‐free system (Figure 2A), treatment with the extract at concentrations ranging from 0.1 to 300 μg/mL resulted in a dose‐dependent increase in dsDNA fluorescence intensity compared with the negative control. However, this assay does not necessarily indicate structural stabilization or absence of DNA damage, as it may reflect alterations in DNA conformation, dye accessibility, or extract–DNA interactions rather than true genomic safety. Therefore, the observed increase should be interpreted cautiously, and additional orthogonal assays, particularly cell‐based approaches such as the comet assay, would be required to comprehensively assess genotoxic safety.

In the genomodulatory assay (Figure 2B), oxidative stress was induced with 5 M H_2_O_2_, which markedly reduced dsDNA fluorescence compared with the untreated control, confirming substantial DNA damage. Co‐treatment with Cascau effectively mitigated this effect in a dose‐dependent manner. Fluorescence intensity increased progressively with extract concentration, and the 300 μg/mL dose restored the signal to more than 300% of the H_2_O_2_ control (*p* < 0.001). These results indicate a robust genoprotective effect, particularly at higher concentrations.

### 3.3 Cascau's Toxicological Effects on *D. melanogaster*

**FIGURE 3:**
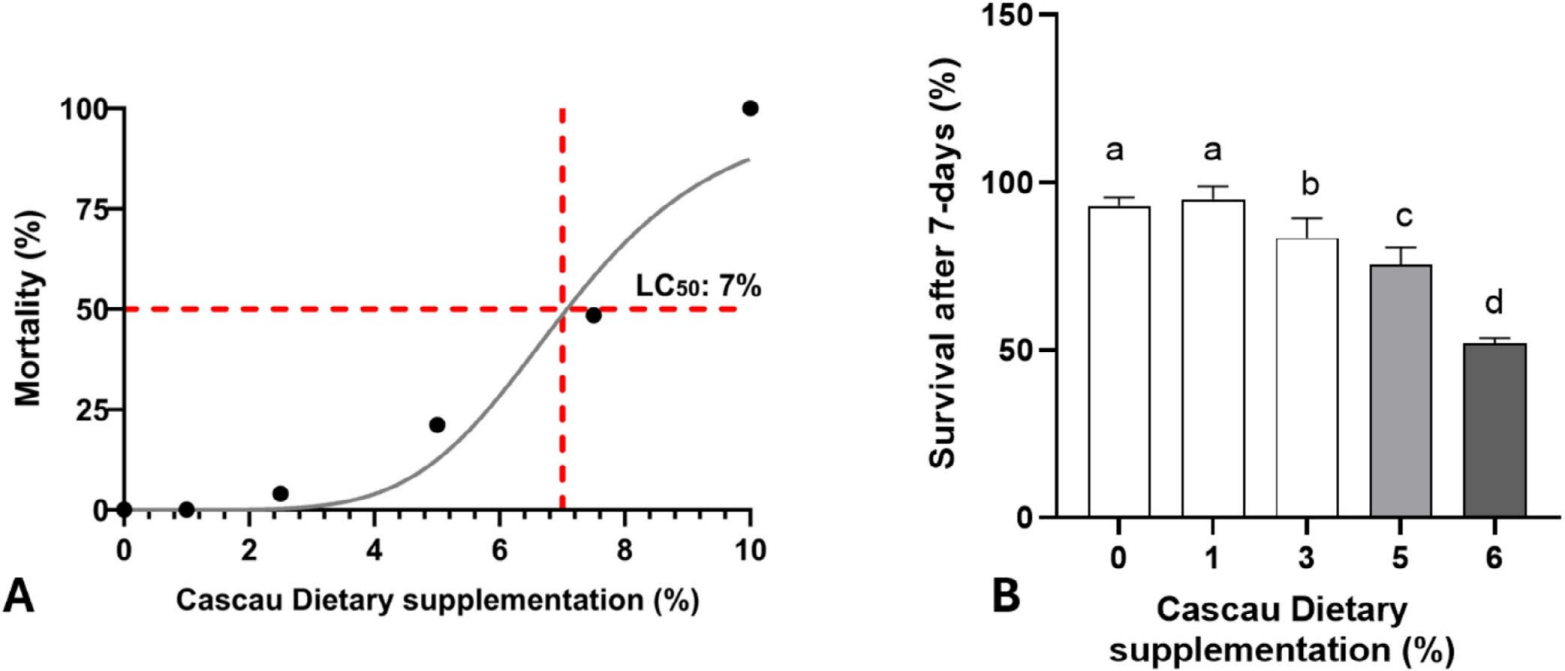
(A) Assessment of the lethal concentration (LC_50_) of Cascau that resulted in 50% mortality of flies after 48 h of exposure to concentrations ranging from 0% to 10% determined by nonlinear regression using the four‐parameter logistic model (4PL). (B) Survival of flies exposed to Cascau at concentrations between 0% and 6% over 7 days. Bar graph values are expressed as mean ± standard deviation (SD) from four independent experiments (*n* = 4). Statistical comparison was performed by one‐way analysis of variance (ANOVA), followed by Tukey's *post hoc* test. Different letters above bars indicate statistically significant differences at *p* ≤ 0.05. Groups sharing the letter “a” are not significantly different from the respective control.

Next, the effects of Cascau on toxicity markers in fruit flies were evaluated. Acute toxicity of the dietary Cascau was assessed in *D. melanogaster* following 48 h of exposure to increasing concentrations (0%–10%). The resulting dose–response curve demonstrated a clear concentration‐dependent increase in mortality (Figure 3A). The median lethal concentration (LC_50_) was estimated at 7% Cascau in the diet, on the basis of a sigmoidal regression model fitted to the data. Mortality remained low at concentrations below 5% but rose markedly at 6% and 10%, indicating a threshold beyond which the extract exerts significant toxic effects. These findings suggest that Cascau may induce acute toxicity when ingested at elevated dietary levels.

To evaluate long‐term toxicity, survival was monitored after 7 days of continuous dietary supplementation with Cascau at concentrations ranging from 1% to 6% (Figure 3B). As illustrated, survival rates declined progressively with increasing extract concentration. Although flies receiving 1% Cascau displayed survival rates comparable to those of the control group, statistically significant reductions were observed from the 3% concentration onwards (*p ≤ 0.05)*.

### 3.4 Cascau Neuro‐Behavioral Effects on *D. melanogaster*

**FIGURE 4:**
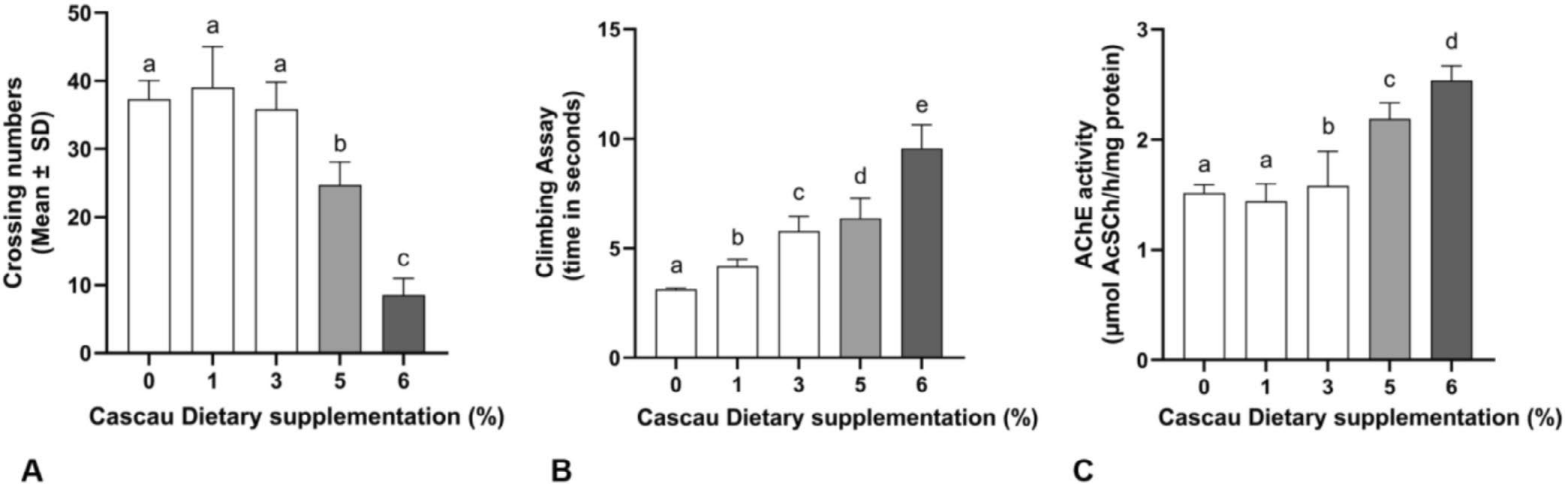
Evaluation of the locomotor activity of *Drosophila melanogaster* exposed to different concentrations of *Cascau* using the following behavioral assays: (A) Open Field Test and (B) Negative Geotaxis. In addition, (C) Acetylcholinesterase (AChE) activity was assessed. Bar graphs represent values as mean ± standard deviation (SD) from four independent experiments (*n* = 4). In both assays, treatments were statistically compared using one‐way analysis of variance (ANOVA), followed by Tukey's *post hoc* test. Different letters above bars indicate statistically significant differences at *p* ≤ 0.05. Groups sharing the letter “a” are not significantly different from the respective control.

The open field assay was used to assess the locomotor activity of *D. melanogaster* following exposure to different concentrations of Cascau (1%–6%). As shown in Figure 4A, supplementation at 1% and 3% did not significantly affect the number of crossings compared to the control group (0%), suggesting no adverse effects on locomotor performance at these concentrations. However, a significant reduction in the number of crossings was observed at 5% (*p* = 0.0012), indicating impaired locomotor function. This effect was even more pronounced at 6%, which exhibited the lowest activity level and a highly significant difference compared to all other groups (*p* < 0.0001). These findings indicate that higher concentrations of Cascau may impair motor performance in *D. melanogaster* , potentially because of neurotoxic or sedative effects.

Motor performance was further evaluated using the negative geotaxis (climbing) assay after dietary supplementation with increasing concentrations of Cascau (1%–6%). As illustrated in Figure 4B, supplementation resulted in a concentration‐dependent decline in climbing ability. Flies exposed to 3%, 5%, and 6% concentrations exhibited significantly prolonged climbing times relative to the control group (*p* < 0.0001), whereas the 1% group did not differ statistically. The 6% group demonstrated the most pronounced deficit, requiring more than 10 s to complete the test. These results suggest that chronic exposure to higher concentrations of Cascau may compromise neuromotor function in *D. melanogaster* , consistent with potential neurotoxic effects.

To investigate possible neurochemical alterations, acetylcholinesterase (AChE) activity was measured following Cascau supplementation. As presented in Figure 4C, AChE activity increased progressively in a dose‐dependent manner starting at the 3% concentration. Although no significant differences were detected at 1% or 3%, activity levels were significantly elevated at 5% and peaked at 6% (*p* = 0.0004 and *p* < 0.0001, respectively). This trend suggests that higher doses of Cascau may modulate cholinergic signaling by enhancing AChE activity, potentially indicative of neurostimulatory or neurotoxic responses depending on the physiological context. These findings further support the hypothesis that chronic exposure to elevated Cascau concentrations can modulate neurochemical pathways in *D. melanogaster* .

### 3.5 Cascau Effects on Oxidative Metabolism Parameters

**FIGURE 5:**
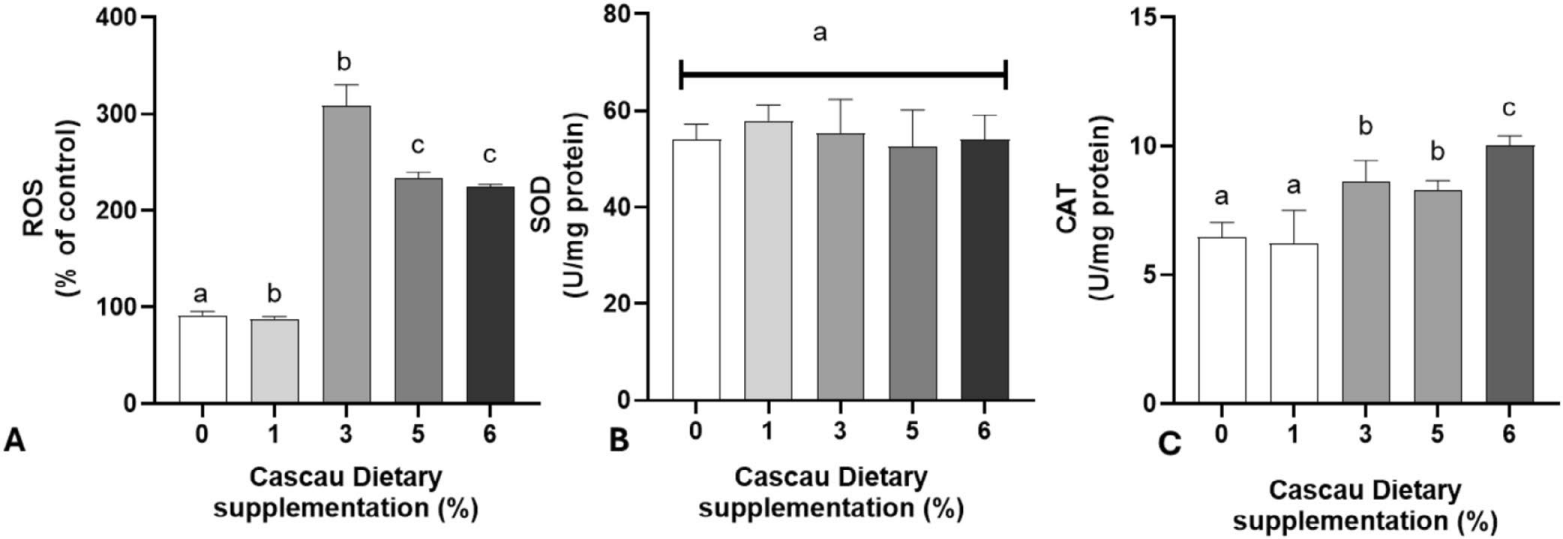
Modulation of oxidative metabolism markers in *Drosophila melanogaster* supplemented with varying concentrations of Cascau (%). (A) Reactive oxygen species (ROS) production and the activity of the antioxidant enzymes (B) superoxide dismutase (SOD) and (C) catalase (CAT). Bar graphs display values as mean ± standard deviation (SD), from four independent experiments (*n* = 4), comparing each Cascau concentration to the control group. Data were statistically analyzed using one‐way analysis of variance (ANOVA), followed by Tukey's *post hoc* test. Different letters above the bars indicate statistically significant differences at *p* ≤ 0.05. Groups sharing the letter “a” are not significantly different from the respective control.

In the quantification of ROS (Figure 5A), flies supplemented with Cascau at concentrations of 3%, 5%, and 6% exhibited significantly elevated levels compared to the control group (*p* < 0.0001). In contrast, no statistical difference was observed between the 1% supplementation group and the control (*p* = 0.9990). Catalase (CAT) activity followed a similar trend, with marked increases observed at 3%, 5%, and 6% concentrations (*p* = 0.0040, *p* = 0.0139, and *p* < 0.0001, respectively). This response likely reflects a compensatory mechanism triggered by elevated hydrogen peroxide (H_2_O_2_) levels, given the key role of CAT in H_2_O_2_ detoxification (Figure 5B). The concurrent increase in both CAT activity and ROS supports the hypothesis of a redox imbalance induced by higher doses of the extract. Superoxide dismutase (SOD) activity, however, remained relatively stable across all groups, suggesting that the generation of superoxide radicals was not substantially altered by Cascau supplementation, or alternatively, that SOD activity may have reached a regulatory plateau under the tested conditions (Figure 5C).

Collectively, these findings suggest that higher concentrations of Cascau disrupt oxidative homeostasis, primarily by increasing intracellular H_2_O_2_ levels. The observed upregulation of CAT, in the absence of changes in SOD activity, further supports the involvement of H_2_O_2_ as the principal reactive species. This redox disturbance may underlie the physiological and behavioral alterations observed in the exposed flies.

## 4 Discussion

The present study evaluated the extent to which dietary supplementation with Cascau could influence toxicity parameters, neurobehavioral responses, and oxidative metabolism in *D. melanogaster* . Although the chemical characterization of Cascau indicated a composition with functional potential, subchronic exposure (7 days) resulted in some undesirable alterations, suggesting that the therapeutic window of Cascau, within which health benefits may be achieved, is relatively narrow. In the following sections, we explore these findings in greater depth and discuss the potential of Cascau as a dietary supplement (Duque 2024).

The chemical characterization of the Cascau confirmed its richness in polyphenols, tannins, and flavonoids, classes of compounds widely recognized for their antioxidant and therapeutic potential (Belwal et al. 2022; Duque 2024). Cocoa bean shells are technically considered a waste product of cocoa processing and pose environmental challenges. However, it is rich in polyphenols, methylxanthines, dietary fiber, and phytosterols, which can be extracted and repurposed in various food and health‐related applications. In this context, various extraction strategies have been explored for the preparation of cocoa bean shell‐based extracts and the recovery of bioactive compounds (Belwal et al. 2022; Hernández‐Hernández et al. 2019).

In the present study, the extract was obtained using a combination of temperature control and pH adjustment, without the use of organic solvents that could pose environmental risks. Despite the simplicity of this extraction method, Cascau exhibited high levels of bioactive constituents, indicative of a robust phytochemical matrix, comparable to, or even surpassing, those reported in other plant‐based preparations (Won and Kwon 2024). LC–MS analysis revealed a chemically diverse profile, with a greater number of compounds detected in negative ionization mode than in positive mode. This finding suggests a predominance of acidic or polar compounds, such as phenolic acids, whereas the positive mode primarily captured flavonoid glycosides and other neutral or basic constituents. The identification of structurally diverse molecules reflects the chemical complexity of Cascau and may help explain the broad range of biological activities observed in subsequent assays. Previous studies have also favored water‐based extraction approaches, yielding chemical matrices with similarities to those of Cascau. For instance, in a heat‐assisted extraction protocol (100°C, 90 min, 0% citric acid, and 0.02 g cocoa shell/mL of water) (Rebollo‐Hernanz et al. 2021), UPLC‐ESI‐MS/MS analysis revealed the presence of 15 phenolic compounds, with protocatechuic acid, procyanidin B2, (−)‐epicatechin, and (+)‐catechin being the major constituents.

On the basis of the presence of bioactive components identified in Cascau—particularly phenolic molecules—its potential antioxidant and genome‐modulating capacities were evaluated using two cell‐free assays: the DPPH and GEMO assays. The antioxidant potential of Cascau was initially assessed through the DPPH assay, where the IC_50_ value indicated a moderate free radical scavenging capacity (Fatmawaty et al. 2019). Although this activity is lower than that of highly purified antioxidant compounds, it remains noteworthy considering the extract's simple aqueous‐based preparation and its complex phytochemical profile. In this context, the presence of phenolic acids and flavonoids likely contributes to the observed activity, albeit in a matrix where synergistic or antagonistic interactions between compounds may influence overall potency.

It is important to acknowledge that the DPPH assay represents a purely chemical radical‐scavenging test and does not reflect cellular antioxidant activity, bioavailability, metabolic transformation, or redox modulation within biological systems. Therefore, these findings should be interpreted as an initial screening of radical‐scavenging capacity rather than direct evidence of intracellular antioxidant efficacy.

The interaction of the Cascau extract with purified double‐stranded DNA (dsDNA) was evaluated in a cell‐free model (GEMO assay). Under the experimental conditions tested, no reduction in PicoGreen fluorescence was observed, indicating that the extract did not promote detectable dsDNA degradation within this simplified system.

However, the increase in DNA‐associated fluorescence should be interpreted with caution. Although this pattern may suggest reduced susceptibility to oxidative degradation, enhanced PicoGreen fluorescence can also result from non‐covalent DNA‐compound interactions, such as minor‐groove binding or intercalation, which may alter dye accessibility without reflecting true structural stabilization. Therefore, these findings do not allow a definitive conclusion regarding the absence of genotoxic effects detectable by this cell‐free assay. Instead, the fluorescence increase may reflect interactions between extract constituents and dsDNA, warranting further mechanistic investigation (Cadoná et al. 2014).

In the genoprotective condition of the GEMO assay, the extract attenuated the fluorescence loss induced by the pro‐oxidant agent, suggesting reduced oxidative damage to dsDNA under cell‐free conditions. However, it is important to emphasize that the GEMO assay represents a non‐cellular system that lacks metabolic activation, chromatin organization, DNA repair pathways, and cellular signaling mechanisms. Therefore, it cannot fully predict genotoxic or genoprotective responses in living organisms. Although the results indicate a potential protective interaction with DNA under simplified experimental conditions, a comprehensive genotoxic evaluation would require complementary cell‐based assays, such as the comet assay or the micronucleus test, to establish biological relevance (Hazafa et al. 2020).

Similarly, in neurodegenerative diseases such as Parkinson's disease, oxidative stress plays a central role in the degeneration of dopaminergic neurons. H_2_O_2_ and other ROS contribute to neuronal damage, mitochondrial dysfunction, and inflammation in the brain. Therefore, compounds capable of neutralizing oxidative species or protecting cellular components, especially DNA, may help delay or reduce neuronal degeneration (Azzolin et al. 2025).

Neurobehavioral assays provided further insight. The integrated analysis of behavioral and enzymatic responses highlights a dose‐dependent neuromodulatory effect of Cascau in *D. melanogaster*. As observed in the climbing and open field, higher concentrations of Cascau (≥ 5%) led to a significant reduction in locomotor performance, indicative of impaired neuromuscular function. This decline coincided with a marked increase in AChE activity, suggesting a disruption in cholinergic signaling. Elevated AChE levels may reduce synaptic acetylcholine availability, contributing to the observed deficits in movement and coordination. Furthermore, antioxidant enzyme activity (likely catalase or SOD) also increased with rising extract concentrations, supporting the notion that Cascau triggers oxidative stress responses. Together, these findings suggest that although the extract may possess neuroactive and antioxidant properties, excessive dosing can induce a state of redox imbalance and neurochemical disruption, ultimately compromising organismal health. Therefore, these effects emphasize the importance of dose optimization, as Cascau appears to have a narrow therapeutic window.

Although most studies indicate that antioxidant‐rich plant extracts are beneficial to fruit flies, some investigations have also reported adverse effects. One such example is the medicinal plant *Croton campestris* (Euphorbiaceae), a species native to Northeastern Brazil, traditionally used to treat a variety of health conditions. However, the hydroalcoholic extract of this plant—also rich in compounds identified in Cascau, particularly caffeic acid and kaempferol—was found to impair locomotor performance and induce mortality in *D. melanogaster* (Júnior et al. 2016). A significant adverse effect of the aqueous stem bark extract of *Mangifera indica* on fruit flies has also been reported (Pam et al. 2021).

A possible explanation for the adverse effects observed with dietary supplementation with Cascau is that its interaction with other components of the standard *D. melanogaster* diet may have resulted in unexpected biological consequences. The standard medium contains complex nutritional elements, including gut microbiota, which play a key role in nutrient metabolism and gut microbial balance in flies. Polyphenol‐rich plant extracts, such as Cascau, are known to exert antimicrobial activity, particularly against fungi and bacteria. It is therefore conceivable that the extract may have disrupted the normal microbiota or impaired the metabolic activity of dietary yeast. Such interactions could indirectly affect nutrient availability, digestion, or microbial symbiosis, leading to physiological stress or nutrient deficiencies that compromise survival and neuromotor performance over time. Moreover, certain phytochemicals can bind to proteins or metal ions in the diet, altering the bioavailability of essential nutrients. These complex interactions are often not evident in cell‐free assays but can significantly influence outcomes in whole‐organism models such as *D. melanogaster* (Del Rio et al. 1999; Pam et al. 2021). Another possible explanation for the observed increase in ROS and catalase activity in *fruit flies* exposed to Cascau lies in the paradoxical redox behavior of polyphenols. Although these compounds are typically regarded as antioxidants, several studies have shown that they can exhibit pro‐oxidant properties under specific physiological conditions. This dual action depends on factors such as concentration, cellular redox state, metal ion availability, and exposure duration.

It is hypothesized that Cascau, being rich in phenolic compounds, may initially trigger a reductive stress, characterized by an excessive electron‐donating environment, which could disrupt redox homeostasis and paradoxically lead to an overproduction of ROS. This redox imbalance can result in the secondary generation of H_2_O_2_, mainly if the extract exhibits vigorous SOD‐like activity, converting superoxide radicals into H_2_O_2_. In turn, this could activate compensatory mechanisms, such as increased catalase activity, which aims to decompose H_2_O_2_ and mitigate oxidative damage. Such a scenario would explain the simultaneous rise in both ROS levels and catalase activity observed in flies exposed to higher concentrations of Cascau. It also reinforces the idea that plant‐derived antioxidants can exhibit context‐dependent behavior and that their biological effects must be evaluated within the complexity of living systems (Del Rio et al. 1999; Galati and O'Brien 2004).

Furthermore, the presence of methylxanthines in cocoa bean shell, particularly theobromine and caffeine, may represent an important contributing factor to the neurobehavioral alterations observed (Daly 2007; Monteiro et al. 2016). These compounds act primarily as antagonists of adenosine receptors and are well known to modulate central nervous system excitability and cholinergic signaling pathways, which may indirectly influence AChE activity. At higher concentrations, methylxanthines have been associated with increased neuronal excitability and potential neurotoxic manifestations (Nehlig et al. 1992; Chen et al. 2013; Rivera‐Oliver and Díaz‐Ríos 2014), which could partially explain the behavioral and enzymatic alterations detected in the present model.

In light of these findings, additional studies employing mammalian models are warranted to further elucidate the translational relevance of these effects and to determine the neurotoxic and cholinergic impact of cocoa bean shell constituents under more physiologically complex conditions.

## 5 Conclusion

Taken together, the findings suggest that Cascau may exert specific functional effects in *D. melanogaster* . However, unlike the roasted cocoa bean traditionally used in chocolate production, the cocoa bean shell appears to exert therapeutic effects within a narrow concentration range, with higher doses inducing adverse effects, possibly related to oxidative stress and neurotoxicity. These results indicate a limited therapeutic window and highlight the importance of dose optimization. Although Cascau exhibits promising bioactivity, its application in functional foods or dietary supplements requires careful evaluation. Further investigations, particularly in mammalian systems and disease‐relevant models, are necessary to validate translational relevance, clarify the contribution of methylxanthines to neurotoxicity, and comprehensively assess safety and bioavailability for human consumption.

## Author Contributions

**Fernanda dos Santos Trombini:** conceptualization, investigation, writing – original draft, resources. **Débora Luísa Pulcinelli:** investigation, writing – original draft. **Nathália Cardoso de Afonso Bonotto:** conceptualization, investigation, writing – original draft. **Caroline Prado Rehbein:** investigation, writing – original draft. **Elize Musachio:** conceptualization, investigation, data curation, writing – original draft, writing – review and editing. **Maria Eduarda Chelotti:** investigation, writing – original draft. **Euler Ribeiro Filho:** data curation. **Eliana Jardim Fernandes:** investigation, writing – original draft. **Fernanda Barbisan:** conceptualization, data curation, writing – review and editing, resources. **Maria Denise Schimith:** conceptualization, data curation, writing – review and editing. **Ivana Beatrice Mânica da Cruz:** conceptualization, data curation, writing – review and editing. **Vitória Azzolin:** investigation. **Verônica Farina Azzolin:** writing – original draft.

## Funding

This work was funded by the Open University Foundation for the Elderly (FUnATI) and Amazonas State Research Support Foundation (FAPEAM). Furthermore, the authors acknowledge the financial support provided by the Coordination for the Improvement of Higher Education Personnel (CAPES) and the National Council for Scientific and Technological Development‐ Universal Project CNPq/MCTI N° 10/2023.

## Conflicts of Interest

The authors declare no conflicts of interest.

## Data Availability

The data that support the findings of this study are available from the corresponding author upon reasonable request.
